# Update on Selected Adverse Events of Concern Following Immunization With Acellular Pertussis Vaccines, a Narrative Review

**DOI:** 10.1111/apa.70676

**Published:** 2026-07-12

**Authors:** Lennart Nilsson, Jann Storsaeter

**Affiliations:** ^1^ Department of Biomedical and Clinical Sciences Linköping University Linköping Sweden; ^2^ Former Allergy Center, now Guest professor at Linköping University Linköping Sweden; ^3^ Former Vaccine Department of the Public Health Agency Stockholm Sweden

**Keywords:** acellular pertussis, adjuvants, adverse events, aluminium, anaphylaxis

## Abstract

**Aim:**

To provide an update paper on evidence of adverse events of concern following immunization (AEFI) with pertussis vaccines, focusing primarily on acellular (aP) vaccines.

**Methods:**

Evidence was synthesised from epidemiological studies, systematic reviews over recent decades, expert interpretations in reviews from relevant research teams and well‐conducted observational reports across different time periods. A more comprehensive review of the literature was conducted for certain specific AEFIs, including sterile abscesses, macrophagic myofasciitis, reactions to aluminium adjuvants, Guillain–Barré syndrome, acute disseminated encephalomyelitis (ADEM) and anaphylaxis.

**Results:**

Acellular (aP) vaccines are generally associated with mild reactions such as local pain, redness, and transient fever. Moderate reactions, including prolonged crying, hypotonic‐hyporesponsive episodes, extensive limb swelling, and aluminium‐induced granuloma, occur infrequently and are self‐limiting. Severe outcomes such as seizures or encephalopathy have been extensively investigated, with large studies showing no consistent increase above background incidence.

**Conclusion:**

Licensed acellular pertussis vaccine combinations demonstrate acceptable safety profiles, with serious adverse events being rare. Continuous pharmacovigilance and balanced, evidence‐based communication remain essential to sustain trust in pertussis immunisation programmes.

## Summary

- This narrative review updates the latest evidence on adverse events of concern following immunization, with a primary focus on acellular pertussis vaccines.
- Licensed pertussis vaccine combinations have acceptable safety profiles. Serious adverse events are rare.
- Continuous pharmacovigilance and balanced, evidence‐based communication remain essential to sustain trust in acellular pertussis immunization programs.

**Abbreviations**
ADEM: acute disseminated encephalomyelitis
AEFI: adverse events following immunisation
DTaP: diphtheria/tetanus/acellular pertussis vaccine
GBS: Guillain–Barrè syndrome
HHE: hypotonic hyporesponsive episode
Hib:
*Haemophilus influenzae* type b vaccine
IgE: immunoglobulin E
IPV: inactivated polio vaccine
SIDS: sudden infant death syndrome
Tdap: tetanus/reduced diphtheria toxoid/acellular pertussis vaccine

## 1 Introduction

Vaccination against pertussis has historically been successfully performed in many countries using whole‐cell pertussis vaccines (wP). These continue to be used in several global regions. Less reactogenic acellular pertussis vaccines were developed in the 1970s and 1980s and their efficacy has been proven in clinical trials. However, from 1974 until today, mistrust of pertussis vaccines has led to low coverage in certain countries and this has led to pertussis rebound, with significant morbidity and mortality [1].

Systematic surveillance for adverse events of concern following immunization (AEFI) was initiated in many countries when acellular pertussis combinations were introduced into infant vaccine programs. Safety signals were reported and followed up by authorities worldwide, including Norway and Sweden. Extensive systematic reviews were published, with later updates by the Cochrane Institute and others [2, 3, 4, 5, 6, 7, 8, 9, 10].

## 2 Aim

The aim of this narrative review was to update the latest evidence on AEFIs for acellular pertussis vaccines.

## 3 Methods

Since 1996 we have on a day‐to‐day basis followed the published literature on this topic and identified relevant publications. Acellular pertussis vaccines are usually administered intramuscularly, in combination with diphtheria and tetanus antigen and often co‐formulated with antigens against *Haemophilus influenzae* type b (Hib), hepatitis B and/or polio. In addition, pneumococcal and meningococcal conjugate vaccines are commonly given simultaneously.

Evidence was evaluated, in average from 70 to 100 publications per year, altogether numbering approximately 2700 publications, including 17 systematic reviews, during the years 1996–2026. Due to published safety concerns we did a more comprehensive review of the literature for certain specific AEFIs, such as sterile abscesses, macrophagic myofasciitis, reactions to aluminium adjuvants, Guillain–Barrré syndrome, acute disseminated encephalomyelitis and anaphylaxis.

The methods used for our narrative review should be valid for mild, moderate, and serious AEFIs of concern but are dependent on local epidemiology and background incidences.

This narrative review also includes input from experts in relevant clinical fields, such as paediatric neurology for seizure evaluation, and other experts for aluminium granulomas.

## 4 Results

### 4.1 Mild Adverse Events

The most frequently reported AEFIs are mild and transient. As they are well described in textbooks and no new mild adverse events have been added in recent years, they will not be further discussed in this paper (see, for example, ref. [2]). There are four levels of mild adverse events:

Level one events are local reactions that resolve within 24–48 h, including erythema at the injection site which occurs in approximately 40%–50% of infants and swelling, warmth or tenderness at the injection site.

Level two events are systemic reactions, namely low‐grade fever, fatigue and general malaise, irritability, particularly in infants and young children, mild fussiness and prolonged crying.

Level three events are gastrointestinal symptoms, such as decreased appetite and nausea or mild vomiting.

Level four events include headaches, which are more commonly reported in older children and adults, mild myalgia, arthralgia, and pain.

Compared to whole‐cell pertussis vaccine, acellular pertussis vaccines have been associated with significantly lower rates and severity of all listed AEFIs except vomiting [2].

### 4.2 Moderate Adverse Events

#### 4.2.1 Fever

Fever, with an elevated body temperature of more than 39.5°C (103.1°F) [11], is reported in approximately one in 100 infants [11] (Table 1).

**TABLE 1:** Acellular pertussis vaccines in infancy and selected AEFI of concern.

Causality Assessment—WHO Classification		Seriousness	Reported incidence/100 000 doses (ref)
Consistent with causal association to immunisation (A) Vaccine related reactions	Mild adv events	Mild	40 000–50 000 [2]
	Fever, > 39.5°C	Moderate	1000 [11]
	Extensive limb swelling	Moderate	Rare in infants^a^ [12]
	Hypotonic hyporesponsive episodes (HHE)	Moderate	4–140 [13]
	Sterile abscess	Moderate	0.18–0.54 [14]
	Persistent crying	Moderate	0–200 [15]
	Discoloured leg syndrome	Moderate	More common after dose 1 and 2, incidence figures are not available
	Aluminium granulomas	Moderate	630 [16]
	Anaphylaxis	Serious	0.2 [17]
Indeterminate (B) Temporal relationship is consistent but there is insufficient evidence for vaccine causing event	Macrophagic myofasciitis	Moderate	Uncertain diagnosis, no valid estimate for incidence [18]
	Febrile seizures	Serious	0.8–8.5 per day first week after 1st to 3rd vaccination [19]^b^
	ADEM	Serious	0.04 [20]^c^

^a^ Extensive limb swelling. Low number after the primary infant series but about 2%–6% after booster doses [12].

^b^ Febrile Seizure was 0.8/100 000 calculated person days for the first vaccination, 1.3/100 000 calculated for the second vaccination and 8.5/100 000 calculated person days for the third vaccination, day 0–7, after DTaP‐IPV‐Hib vaccinations [19].

^c^ Uncertain incidence based on only two cases in one publication [20]. No evidence of an association in children under 7 years in a pilot study, Australia [21].

#### 4.2.2 Extensive Limb Swelling

Extensive limb swelling, namely a large localised swelling around the injection site, has often been associated with booster doses of DTaP. This AEFI was further evaluated by Rennels and Edwards [12], who provided some evidence that high immunoglobulin G antibody levels before boosters might play a causative role, particularly with regard to diphtheria toxoid [2].

#### 4.2.3 Hypotonic Hyporesponsive Episodes (HHE)

Hypotonic hyporesponsive episodes (HHE) are defined by sudden onset of pallor or cyanosis, limpness and reduced responsiveness, within 48 h of vaccination. Infants under 6 months of age are at the highest risk and the median age of onset is 4 months (range 1.1–107 months) [22]. One study reported that the median interval between vaccination and hypotonic hyporesponsive episodes was 210 min (range 1 min to 2 days) [22]. Other studies reported that HHE has a duration of 6–30 min, with spontaneous recovery and no association with long‐term neurological or developmental sequelae [23]. Affected infants might often remain drowsy for 24–48 h [24]. Eighty percent of HHE cases occurred after the first vaccine dose, 18% after the second dose and only rarely after the third dose [24]. The exact pathophysiology for HHE remains unclear, but it is thought to involve an autonomic dysregulation or immune‐mediated response. HHE have been estimated to occur in approximately 4–140 per 100 000 doses of acellular pertussis vaccines in infants [13] (Table 1). From 1996 to 1998, the number of hypotonic hyporesponsive episodes reports from the USA Vaccine Adverse Event Reporting System decreased in 145 to 81 per 100 000. This was when the predominant pertussis vaccine administered to infants changed from whole‐cell to acellular [22]. Parental education is essential, with the emphasis on the benign nature of these episodes.

#### 4.2.4 Sterile Abscesses at Vaccination Sites

We found seven recent (1996–2026) publications on this subject. Sterile abscesses typically present as firm, round subcutaneous swellings, often without accompanying erythema, warmth, or pain. The estimated incidence following DTaP and Tdap vaccines has been quantified as approximately 0.18–0.54 cases per 100 000 doses [14] (Table 1). They have been reported to appear 1 day to several weeks, mean 15 days, after vaccination [25]. The primary etiological factor implicated in these AEFIs is the aluminium‐based adjuvants used in vaccines, including aluminium phosphate and aluminium hydroxyphosphate sulphate [25]. Most sterile abscesses resolve spontaneously without intervention, and antibiotic therapy is not indicated, given their sterile nature. Sterile abscess formation does not contraindicate future vaccinations but can recur after subsequent vaccinations [25].

#### 4.2.5 Persistent Crying

Persistent crying is characterized by episodes of inconsolable, high‐pitched crying, lasting for 3 h or more within 48 h of vaccination. Although distressing, persistent crying is self‐limiting, generally resolves spontaneously and has not been associated with long‐term health consequences. The exact mechanism involved is unclear, but hypotheses include local pain, systemic inflammatory responses and transient neurological irritation associated with vaccine reactogenicity. A randomised trial by Gustafsson et al. [15] found that persistent crying that lasted for at least 3 h occurred in 0%–0.2% of infants who received acellular pertussis vaccines (Table 1) and 1.1%–1.2% for whole‐cell pertussis vaccine recipients.

#### 4.2.6 Discoloured Leg Syndrome

Discoloured leg syndrome is defined as an AEFI with even or patchy red, blue or purple discolorations of one or both legs and/or leg petechiae, with or without swelling. It most commonly manifests after the first or second vaccination dose, but does not radiate from the injection site. The syndrome has been reported to occur homolaterally (27%), contralaterally (2%), on both sides (64%) or unspecified (7%) [26]. Studies also show that 80% of discoloured leg syndrome cases occur after combination vaccines, specifically DTaP‐IPV + Hib. It is frequently accompanied by intense, inconsolable crying (78%). The median time from vaccination to discoloration is reported as 3.8 h and the median duration is short, lasting approximately 2 h, with wide variations. Furthermore Dutch investigators reported that petechial bleeding was more common below 5 months of age, most frequently noticed within 2 days and disappeared within 1 week [27]. Discoloured leg syndrome episodes are self‐limiting and resolve without intervention. It has been hypothesised that this AEFI may be caused by a flushing or vasomotor reaction. Clinicians should be aware that this syndrome, accompanied by inconsolable crying, has been reported to have a major impact on parental anxiety [26].

#### 4.2.7 Macrophagic Myofasciitis

Macrophagic myofasciitis is a rare AEFI, which was first described in 1998 [28]. We found 21 recent (1996–2026) publications on this subject. It is an inflammatory myopathy, characterized by persistent myalgia or arthralgia and distinctive inflammatory infiltrates in muscle biopsies. Histologically these lesions have been marked by aggregates of macrophages containing aluminium salts, often found at previous vaccination injection sites. The microscopic characteristics of macrophagic myofasciitis can be recognised as a physiological, not necessarily pathological reaction, to vaccines containing aluminium hydroxide [29]. Lach et al. [30] described this AEFI as a probable localized histological hallmark of previous immunisation with aluminium adjuvants contained in vaccines, rather than as a primary or distinct inflammatory muscle disease. Despite worldwide childhood immunisation programs, only a few paediatric cases of macrophagic myofasciitis have been reported [18].

#### 4.2.8 Aluminium Salts

Including aluminium hydroxide and aluminium phosphate, have been used as adjuvants in vaccines for more than 90 years. The adjuvants enhance immunogenicity by promoting antigen uptake and stimulating a robust lasting immune response. Aluminium is regarded as an essential component of many paediatric vaccines including all marketed acellular pertussis vaccines even though the mechanisms behind its biological beneficial effects are not fully understood. There has been published concerns that aluminium may be linked to developmental, neurologic, allergic and/or autoimmune disease and thorough reviews have recently (March 2026 and April 2026) been published in Paediatrics [31] and British Medical Journal [10]. In addition, we found 143 recent publications on this subject during the years 1996–2026.

#### 4.2.9 Aluminium Pharmacogenetics

While aluminium is ubiquitously present in the environment, such as in food, water and air, pharmacokinetics vary substantially, depending on the route of exposure [32]. Following parenteral administration via vaccination, aluminium is gradually released from the injection site into systemic circulation. The majority is excreted renally, with limited accumulation in soft tissues due to its short biological half‐life of less than 2 days. Long‐term storage primarily occurs in the skeletal system, where aluminium can persist for years [33]. Aluminium levels are lower in the brain and central nervous system (1%) than in many other body tissues, such as in the liver and spleen [34].

#### 4.2.10 Aluminium Toxicity

Aluminium toxicity has been a concern. High levels of aluminium have been associated with encephalopathy, bone disease and anaemia in dialysis patients; after industrial exposure and in animal studies [35]. These high levels have not been shown after vaccination, as their dosage is too low to create toxic effects. Goullé and Grangeot‐Keros emphasize that systemic aluminium levels remain within physiological ranges after vaccination, even in infants. They state that they did not differ significantly from levels observed in unvaccinated individuals [35]. Another study showed that the transient elevation of aluminium after vaccination did not approach toxic thresholds, not even in infants or low birth‐weight neonates [32].

#### 4.2.11 Allergy

Concerns about potential links between aluminium adjuvants and allergic diseases, particularly about asthma and atopy, have been voiced, but large‐scale epidemiological studies have not substantiated these fears [10, 31]. Aluminium enhances immunogenicity in general and strengthens the immature skewed Th2 response found in infants in the Western hemisphere [36]. However, longitudinal studies from the UK, Sweden and Australia found no association between whole cell or acellular pertussis vaccinations and the development of early‐onset or late‐onset wheezing or recurrent or intermittent wheezing episodes [37, 38, 39]. In Australia, researchers hypothesised that children who received one or more doses of whole cell pertussis vaccine had a lower risk of developing IgE‐mediated food allergy compared with acellular pertussis vaccinated children only [40]. Therefore, they have initiated a prospective randomized study, initially with two arms of 75 children each, to obtain a scientific answer to this hypothesis with clinical and immunological outcomes. The data so far published show no clinically significant differences between arms except for more mild vaccine reactions after whole cell vaccines [41].

A German prospective cohort study by Grüber et al. monitored 943 children up to 5 years of age and systematically assessed the prevalence of atopic symptoms and diagnoses and total and specific IgE levels for nine common allergens. No positive associations were observed between the cumulative number of vaccine doses and the incidences of atopic diseases or allergic sensitization [42]. A Swedish study also found no differences in asthma medication in teenagers who had, or had not, received acellular pertussis vaccines in infancy [43]. Another extensive US cohort study of 167 240 children found no associations between DTwP, polio, mumps or rubella vaccines and asthma [44].

However, a recent large retrospective observational study from the USA, which used data from the Kaiser Permanente Network, suggested a possible link between persistent asthma and aluminium exposure from vaccines [45]. This 2023 paper described a statistically significant association between cumulative aluminium content in vaccines given before 24 months of age and persistent asthma up to 59 months of age [45]. Confounding factors, such as breast feeding or food intake, could have influenced the results, but were not evaluated. Moreover, the study did not show any clear dose–response connection and the risk of selection bias was noted in an accompanying commentary. The results are inconclusive but warrant additional research [46]. One Danish retrospective register study published in July 2025 [47] did not confirm the results of the 2023 paper [45]. The Danish study was in its turn criticized for methodological weaknesses and other limitations, but defended by the editor‐in‐chief, reinforcing the benefit of vaccination. She stated firmly that the study was among the strongest datasets ever to address whether there was an association between the cumulative dose of vaccine‐associated aluminium exposure and chronic conditions in early childhood [47].

#### 4.2.12 Aluminium‐Induced Granulomas

Aluminium‐induced granulomas with persistent nodules and severe itching have also been cause for concern. Aluminium contact allergy has been recognised since the 1940s and granulomas at the injection site, following aluminium‐adsorbed vaccines, were first reported in the 1960s. In 1980, a positive patch test using Finn Chambers confirmed an association between aluminium contact allergy and granuloma formation [48]. Itching granulomas have been increasingly documented in Sweden since the 1990s, following immunization with aluminium‐adsorbed vaccines. A large paediatric cohort study by Bergfors et al. was pivotal in characterizing this AEFI [16]. The investigators estimated that the incidence was 0.63% in children vaccinated with DTaP (Table 1) [49] and 1.18% when pneumococcal conjugate vaccines were administered simultaneously. Itchy aluminium‐induced granulomas may appear months after vaccination, but they are usually benign, self‐limiting and do not contraindicate further vaccinations against serious infectious diseases. The median duration of itching is reported as 6.6 years (range 0.04–19.2 years) [50]. Notably, aluminium granulomas have been mistaken for malignancies, leading to unnecessary investigations, such as ultrasounds, biopsies or surgical excision under anaesthesia [51].

### 4.3 Serious Adverse Events

Vaccine‐related febrile seizures are defined as seizures within 72 h of vaccination. The outcomes are similar to other febrile seizures, which means that further vaccinations can usually be administered [19, 52, 53]. DTaP‐IPV + Hib vaccinations have been associated with an increased risk of febrile seizures in infancy, but studies show that the absolute risk is small [19] (Table 1).

A systematic review from 2022 found no evidence that vaccinations cause epilepsy in healthy populations [53]. However, the authors maintained that vaccination may act as a non‐specific trigger for seizures in some individuals with underlying structural or genetic etiologies. The need to administer further vaccinations under enhanced medical supervision should, in such cases, be considered.

If vaccine‐proximate status epilepticus should occur, children should be evaluated for a possible underlying genetic cause of convulsions.

#### 4.3.1 Withholding Vaccines

Withholding vaccines due to suspected or diagnosed neurodevelopment disorders is important. Current recommendations state that vaccinations should not be administered to patients with progressive neurological disorders, including infantile spasms, uncontrolled epilepsy, or progressive encephalopathy. Vaccination with DTaP combinations should be deferred until the neurological status has been clarified and stabilised [2]. Deng et al. [54] have succinctly summarized a number of key points. Children with a personal or family history of febrile seizures or epilepsy should, as a rule, receive all routine vaccinations. Young infants, particularly those under 12 months of age, with febrile status epilepticus following vaccination could have an underlying genetic epilepsy, such as Dravet syndrome [55, 56]. The first seizure in Dravet syndrome may be earlier in vaccinated children than in non‐vaccinated children, but developmental outcomes have been shown to be similar in both groups [56]. Children with Dravet's syndrome should be referred to a specialist immunization clinic or neurologist for further investigations and advice.

#### 4.3.2 Guillain–Barré Syndrome (GBS)

Guillain–Barré syndrome (GBS) is a rare acute, immune‐mediated polyneuropathy in which the immune system attacks peripheral nerves. We found 26 recent publications on GBS and vaccination. Its incidence has been quantified as 0.6 per 100, 000 in children [57, 58], but more rare in children under 2 years of age [59]. The classic presentation is weakness that is symmetric, progressive and ascending. Although it commonly starts in the lower extremities, weakness can involve the arms and cranial nerves over time. Deep tendon reflexes are diminished or absent on presentation. Pain can be a prominent symptom, especially in young children. In the acute phase, typically within the first 2 weeks of disease onset, patients are at risk of developing complications and extensive nerve damage. Intravenous immunoglobulin and plasma exchange have been shown to be effective in accelerating recovery [60].

Guillain–Barré syndrome (GBS) is believed to be related to infection, with approximately two‐thirds of patients presenting with preceding gastrointestinal or respiratory infections. Many specific pathogens such as *Campylobacter jejuni* , *Haemophilus influenzae* , and influenza A virus have been reported to be associated with GBS [61]. There have been occasional reports of cases after vaccination with old versions of rabies, influenza, and combined DTaP vaccines [62].

There is a regulatory precaution for patients with GBS advising against further tetanus vaccines without considering the patient's history, but the attributable excess risk seems to be negligible for vaccination 6 weeks or more after onset of GBS [61, 62, 63].

#### 4.3.3 Acute Disseminated Encephalomyelitis (ADEM) and Transverse Myelitis

Acute disseminated encephalomyelitis (ADEM) and transverse myelitis are rare inflammatory demyelinating disorders of the central nervous system with relatively acute onset. Some case reports have suggested that vaccines may trigger these disorders. We found 15 recent publications on this topic from 1996 to 2026. Transverse myelitis presents with sudden onset of neurological symptoms, and ADEM with fever and headache. ADEM has an estimated basic incidence of 0.6/100 000 according to Orphanet [64]. Symptoms are rapidly manifested due to brain or spinal cord inflammation. A large‐scale US study by Baxter et al. [20] examined possible associations between vaccination and demyelinating events, using a case‐centred design. The study population included all children and adults in six Vaccine Safety Data Link sites who received one or more vaccines between January 2007 and December 2012. A neurologist with expertise in demyelinating illnesses reviewed each case. The investigation used two exposure intervals: 5–28 days, as this was the most likely interval to result in a demyelinating illness after immunisation, and also 2–42 days. All administered vaccines were evaluated. There was no statistically significant increased risk for transverse myelitis from any single vaccine, but there were two ADEM cases, and one had received multiple vaccines. The Tdap vaccine was associated with borderline significant increased risk for the two cases in the 5 to 28‐day exposure interval (odds risk 15.8, 95% confidence interval 1.2–471.6, *p* = 0.04) but not in the 2 to 42‐day interval. The estimated possible increased risk of ADEM was 0.4 cases per one million doses of Tdap vaccination, not likely to exceed 1.16 cases per million doses of Tdap [20], (Table 1).

However, a retrospective regional study from Nanchang, China and a pilot self‐controlled case series study concerning pre‐school children in Victoria, Australia have not indicated excess risk after vaccination [21].

#### 4.3.4 Anaphylaxis

Anaphylaxis after vaccines containing acellular pertussis is another serious adverse event. We found 42 publications on this topic from 1996 to 2026. Serious and life‐threatening anaphylactic reactions after having received acellular pertussis vaccine have been estimated as 2.1 cases per million doses for DTaP (Table 1) (95% confidence interval 0.4–6.1), and 2.9 cases per million doses for Tdap (95% confidence interval 1.3–5.5), respectively, based on the Brighton Collaboration definition levels 1 and 2 [17]. Although the potential for severe allergic reactions is rare, all vaccination clinics must be prepared with epinephrine/adrenaline to treat anaphylactic reactions immediately.

The reasons for anaphylaxis reactions to vaccinations are usually unknown. Vaccine‐protective antigens per se are seldom, or never, a primary cause of anaphylaxis. Instead, allergic hypersensitivity IgE reactions in real cases may be linked to other specific vaccine constituents. We mention two examples from recent history:

The first is anaphylaxis due to gelatine. A Japanese study found a causal relationship between vaccination with a DTaP containing gelatine in infancy and the risk of anaphylaxis in subsequent immunizations with live viral vaccines that contained large amounts of gelatine as stabilizers [65] before the year 2000. Many well‐documented anaphylaxis cases have been reported after vaccinations with vaccines containing milligrams of gelatine, mainly measles, mumps and rubella vaccines and varicella vaccine [66].

The second example is anaphylaxis due to casein. Kattan et al. [67] reported eight US children with severe cow's milk allergies and anaphylactic reactions after having received DTaP or Tdap vaccines in the US during the years 2007–2010. The vaccines were processed in a medium containing casamino acids. The authors were concerned that residual casein in the vaccines may have triggered these reactions. However, they were unable specifically to test vaccine batches associated with the reactions. Slater et al. raised concerns about the laboratory methods used for detecting casein in vaccine preparations, suggesting the need for further validation of the assays [68]. Anaphylaxis due to casein in pertussis vaccines has not been reported in Sweden to our knowledge.

#### 4.3.5 Mortality in Infancy and Childhood

Mortality in infancy and childhood is another concern. Historically, concerns have been raised about possible associations between acellular pertussis vaccination and sudden infant death syndrome (SIDS). The origin of these concerns has largely been attributed to the temporal overlap between the peak incidence of SIDS and first vaccination in the infant immunization schedule.

The authors who contributed in Plotkin's Vaccines [2] pointed out that SIDS peaks between 2 and 3 months of age, which is when infants are receiving a relatively large number of recommended vaccinations [2].

Data from Sweden and Italy in the 1990s showed that among 98 215 which took part in acellular pertussis vaccinating studies there was a lower incidence of SIDS than among 41 630 vaccinated infants who did not take part. However, the studies did not evaluate for confounding factors, such as whether the infants were placed on their backs to sleep, rather than in the prone face‐down position [69, 70]. A large case‐control study from Germany showed no increased risk for sudden unexpected death with hexavalent pertussis‐containing vaccine; rather, a possible protective effect [71].

#### 4.3.6 Other Non‐Specific Serious AEFI:s Linked With Live and Non‐Live Vaccines

Other non‐specific serious AEFI:s linked with live and non‐live vaccines also need to be considered. A series of studies from Aaby's Danish team in Guinea–Bissau described mortality after vaccination with live and non‐live vaccines [72]. The investigators linked their findings with other published data to put forward three important statements. First, the live measles vaccine reduced non‐target infections [73]. Second, non‐live vaccines enhanced the susceptibility of female infants in poor countries to non‐target infections [73]. Third, the DTP vaccine induced immunotolerance to unrelated antigens [74].

Aaby's et al. studies indicated significant reductions in child mortality over time following vaccination campaigns [75]. However, concerns were raised about unexpected mortality patterns, particularly among girls following DTP vaccinations. It was difficult to interpret all the observations while accounting for confounding socio‐economic and healthcare access variables. Based on their initial findings, the researchers conducted further investigations to identify subgroups of children at increased risk of mortality in urban Guinea–Bissau [76], by using data from the Bandim Health Project, which was a health and demographic surveillance system covering 2003–2019. The study analysed baseline characteristics collected before the children reached 6 weeks of age to identify factors associated with elevated mortality up to 3 years of age.

This follow‐up analysis found three distinct high‐risk subgroups and provided adjustable mortality risk differences with 95% confidence intervals. The first subgroup was children living in a specific high‐risk urban area (3.4%, 0.3%–6.5%). The second was children born to mothers who did not receive prenatal consultations (5.8%, 2.6%–8.9%). The third subgroup was children from polygamous families born during the dry season (1.7%, 0.4%–2.9%).

Although the subgroups were small, they consistently exhibited higher mortality risks over time. These findings underscore the need to contextualise vaccine safety data within a broader framework of public health determinants. Rather than attributing mortality to vaccination per se, the authors suggested complex interactions between socioeconomic status, healthcare access and environmental factors. They advocated future research focusing on developing, testing, and evaluating targeted intervention strategies to mitigate child mortality in high‐risk populations [76].

## 5 Further Discussion

The findings of this updated narrative review underline the need for further research and pharmacovigilance.

Continued efforts are needed to address vaccine hesitancy, by providing transparent, evidence‐based communication and countering misinformation with respectful and fact‐based dialogues. Strengthening public trust in vaccination programs remains a critical priority to maintain high immunisation coverage and ensure population health.

Vaccines save lives. But that is not their only benefit. When health improves, poor countries can spend more on schools, roads and other investments that drive growth, which makes them less dependent on aid. One study quoted in ‘Plotkin's Vaccines’ showed that low‐income and middle‐income countries saw a return of 16–44 US dollars for every US dollar invested in vaccines [2].

Pharmacovigilance plays a central role in safeguarding public health by continuously monitoring the safety of vaccines, especially in an era marked by vaccine hesitancy. It involves the detection, assessment, understanding, and prevention of AEFIs, ensuring that the benefits of vaccines outweigh potential risks. Compared to acellular pertussis vaccines, whole‐cell pertussis vaccines have been shown to cause more frequent local and systemic reactions. However, they provide longer‐lasting protection and remain favourable in settings with a high disease burden.

## 6 Conclusion

Acellular pertussis vaccines have generally been associated with mild reactions, such as local pain, redness, and transient fever. Moderate reactions, including prolonged crying, hypotonic‐hyporesponsive episodes, extensive limb swelling, and aluminium‐induced granuloma, have occurred infrequently and have been self‐limiting. Clinical syndromes such as seizures or encephalopathy have been extensively investigated, with large studies showing no consistent increase above background incidence. ADEM is the only demyelinating disorder with a possible but unconfirmed association.

The benefits of pertussis vaccines massively overwhelm the adverse events linked to vaccination.

## Author Contributions

**Jann Storsaeter:** writing – review and editing, methodology, validation, supervision, investigation, visualization, resources. **Lennart Nilsson:** writing – original draft, methodology, investigation, validation, writing – review and editing, project administration, conceptualization, resources.

## Funding

The authors have nothing to report.

## Conflicts of Interest

The authors declare no conflicts of interest.

## Data Availability

Data sharing not applicable to this article as no datasets were generated or analysed during the current study.
